# FOCUS-MUSE DWI outperforms MUSE and FOCUS DWIs in orbital imaging quality and staging thyroid-associated ophthalmopathy

**DOI:** 10.1186/s13244-025-02129-9

**Published:** 2025-11-06

**Authors:** Lu Chen, Song Gao, Quan Yuan, Ying Wan, Jin-Ling Lu, Jiang Zhou, Huan-Huan Chen, Xiao-Quan Xu, Fei-Yun Wu, Hao Hu

**Affiliations:** 1 Department of Radiology, The First Affiliated Hospital with Nanjing Medical University, Nanjing, China; 2 Department of Radiology, The People’s Hospital of Danyang, Danyang, China; 3 Department of Endocrinology, The First Affiliated Hospital with Nanjing Medical University, Nanjing, China

**Keywords:** Thyroid-associated ophthalmopathy, Diffusion-weighted imaging, Magnetic resonance imaging, Image quality, Staging

## Abstract

**Objectives:**

To compare field-of-view optimized and constrained undistorted single-shot (FOCUS), multiplexed sensitivity-encoding (MUSE) and FOCUS-MUSE diffusion-weighted images (DWIs) in orbital imaging quality and staging performance for the patients with thyroid-associated ophthalmopathy (TAO).

**Materials and methods:**

67 TAOs underwent FOCUS, MUSE and FOCUS-MUSE DWIs. Qualitative (artifacts and geometric distortion, overall image quality, sharpness of boundaries) and quantitative parameters (geometric distortion ratio (GDR), signal-to-noise ratio (SNR), apparent diffusion coefficient (ADC) value, normalized ADC (nADC) value) were assessed. Additionally, nADC values of the extraocular muscles (EOMs) and mean nADC values were compared between active and inactive TAOs. Diagnostic performance was also evaluated.

**Results:**

FOCUS-MUSE DWI exhibited significantly fewer artifacts and geometric distortion, superior overall image quality, enhanced sharpness of boundaries, higher SNR and lower GDR than MUSE and FOCUS DWIs (all *p* < 0.05). FOCUS-MUSE DWI showed significantly lower ADC values than MUSE (all *p* < 0.05) and FOCUS DWIs (all *p* < 0.05, except for that of superior EOM). The nADC values showed no significance among the three DWIs (all *p* > 0.05), except for that of the superior EOM. Furthermore, active TAOs showed higher nADC values than inactive TAOs in three DWIs (all *p* < 0.05). The mean nADC value of FOCUS-MUSE DWI (AUC, 0.890; sensitivity, 84.8%; specificity, 77.3%) performed better than that of MUSE (AUC, 0.713; sensitivity, 54.3%; specificity, 80.7%; *p* < 0.001) and FOCUS DWIs (AUC, 0.730; sensitivity, 47.8%; specificity, 90.9%; *p* < 0.001) in diagnosing active TAOs.

**Conclusions:**

FOCUS-MUSE DWI provides superior image quality and staging performance in assessing TAO than MUSE and FOCUS DWIs. We recommend its use for evaluating TAO patients in clinical practice.

**Critical relevance statement:**

Field-of-view optimized and constrained undistorted single-shot multiplexed sensitivity-encoding DWI shows superior image quality and staging performance for thyroid-associated ophthalmopathy than other echo-planar imaging-based modified sequences.

**Key Points:**

- The superiority among different echo-planar imaging-based modified DWIs in thyroid-associated ophthalmopathy remains unclear.
- Field-of-view optimized and constrained undistorted single-shot multiplexed sensitivity-encoding (FOCUS-MUSE) DWI outperforms MUSE and FOCUS DWIs in imaging quality.
- Normalized apparent diffusion coefficient values derived from FOCUS-MUSE DWI improve staging performance of thyroid-associated ophthalmopathy.

**Graphical Abstract:**

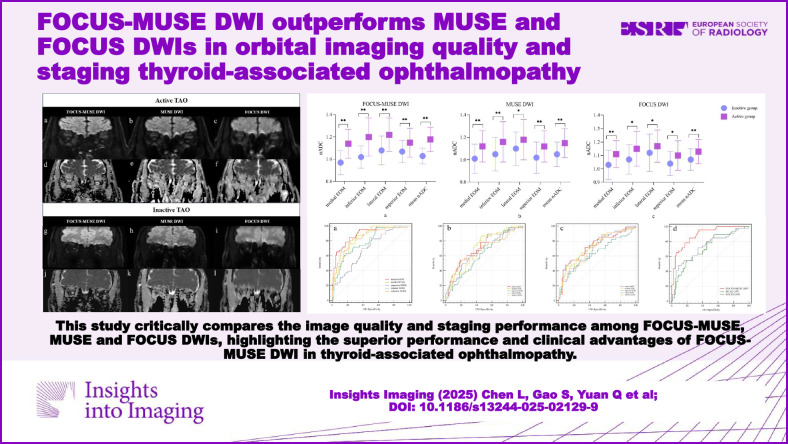

## Introduction

Thyroid-associated ophthalmopathy (TAO) is an autoimmune disorder characterized by inflammation of orbital tissues [1]. The management of TAO depends on accurate assessment of disease activity [1, 2]. Intravenous corticosteroids are commonly used to reduce inflammation in active TAO patients, whereas surgical interventions are possibly reserved for patients with inactive disease [2, 3]. Therefore, accurate evaluation of TAO activity is essential for devising an appropriate therapeutic strategy.

Clinical activity score (CAS), which evaluates TAO activity based on clinical symptoms, is inherently subjective and fails to reflect the involvement of retrobulbar tissues [4]. MRI enables a comprehensive evaluation of the orbit. The signal intensity ratio (SIR) of extraocular muscles (EOMs) derived from T2-weighted imaging (T2WI), a widely used indicator, has been reported to be useful in grading disease activity [5]. However, this semi-quantitative parameter prevents an accurate evaluation of EOM.

Recently, diffusion-weighted imaging (DWI) has been increasingly applied for evaluating TAO [4, 6]. Kilicarslan et al have demonstrated that elevated ADC values of EOMs in patients with TAO may serve as an early diagnostic indicator of the disease [7]. However, the most commonly used conventional DWI sequence in clinical practice, single-shot echo-planar imaging (SS-EPI), is prone to low signal-to-noise ratio (SNR), increased susceptibility artifacts and increased blurring effects of images, resulting in limited diagnostic accuracy [8, 9]. Hence, advanced DWI techniques are needed in clinical practice.

Field-of-view (FOV) optimized and constrained undistorted single-shot DWI (FOCUS DWI), multiplexed sensitivity-encoding DWI (MUSE DWI) and field-of-view optimized and constrained undistorted single-shot multiplexed sensitivity-encoding DWI (FOCUE-MUSE DWI) are EPI-based modified DWIs developed by GE Healthcare Company [10, 11]. Compared to SS-EPI DWI, FOCUS DWI contributes to the reduced image distortion and better anatomical details. However, SNR in reduced FOV DWI may be compromised [12]. MUSE DWI can minimize susceptibility artifacts and improve SNR, which has been used for the evaluation of breast, liver and pancreas [13–15]. By integrating small-field local excitation with interleaved phase-encoding acquisitions, FOCUS-MUSE DWI may perform more uniform signal intensity (SI) and higher image quality than FOCUS DWI and MUSE DWI in theory [13]. Yet, a systematic evaluation of the EPI-based modified DWI sequences of TAO patients has never been performed.

Therefore, the purpose of our study was to systematically compare FOCUS DWI, MUSE DWI and FOCUS-MUSE DWI in orbital image quality and staging performance for the patients with TAO.

## Materials and methods

### Patients

This prospective study was approved by the Ethics Committee of The First Affiliated Hospital with Nanjing Medical University (No. 2022-SR-288), and informed consent was obtained from each patient before MRI scanning. A total of 67 consecutive patients (mean age, 45.0 ± 13.9 years; male/female ratio, 24/43), clinically diagnosed with TAO according to Bartley’s criteria [16], were enrolled between January to September 2024. The inclusion criteria include: (1) concurrent availability of pretreatment orbital DWI sequences, including coronal FOCUS-MUSE DWI, FOCUS DWI and MUSE DWI; (2) adequate image quality for further analysis; 3) bilateral eyes involved; 4) no history of radiotherapy or surgical treatment; 5) no other orbital disease. The exclusion criteria include: (1) incomplete coronal FOCUS-MUSE DWI, FOCUS DWI and MUSE DWI images; (2) poor image quality unavailable for evaluation; (3) prior treatment before MRI scanning, including radiotherapy and surgery; (4) coexisting other orbital diseases, such as orbital tumors.

Disease activity for each eye was evaluated using the modified seven-point CAS proposed by Mourits et al [17], which includes: spontaneous retrobulbar pain, pain on attempted vertical gaze, eyelid redness, conjunctival redness, eyelid swelling, caruncular and/or plical inflammation, and conjunctival edema. Eyes with a CAS ≥ 3 were classified as active, otherwise as inactive. All patients were assessed by an experienced endocrinologist (with over 20 years of experience). Finally, a total of 23 patients with TAO with 46 eyes were defined as active and 44 patients with TAO with 88 eyes as inactive.

### Image acquisition

MRI scans were performed on a 3.0-T system (SIGNA Premier, GE Healthcare, USA) with a 48-channel head coil. Patients were instructed to rest in the supine position with eyes closed to minimize motion artifacts. Detailed imaging parameters of three coronal DWI sequences (FOCUS-MUSE, MUSE, and FOCUS) were summarized in Table 1. Conventional imaging protocols included axial T1-weighted imaging (repetition time (TR)/echo time (TE), 679/10 ms), axial and coronal T2-weighted imaging (T2WI) (TR/TE, 3223–4232/102 ms).

**Table 1:** Imaging parameters of FOCUS-MUSE DWI, MUSE DWI and FOCUS DWI

	FOCUS-MUSE	MUSE	FOCUS
TR (ms)	4500	4500	4500
TE (ms)	Minimum	Minimum	Minimum
Number of sections	18	18	18
Section thickness (mm)	3.5	3.5	3.5
Field of view (cm)	18 × 9	18 × 18	18 × 9
b values (s/mm^2^)	0, 1000	0, 1000	0, 1000
Acquisition time (min:s)	3:09	3:18	1:35

*DWI* diffusion-weighted imaging, *TR* repetition time, *TE* echo time

### Image analysis

Qualitative and quantitative assessments were conducted using *b* = 1000 DWIs and corresponding ADC images. Qualitative measurements of DWI sequences include: (1) artifacts and geometric distortion; (2) overall image quality; (3) sharpness of boundaries (superior, inferior, medial, and lateral EOMs of each eye were evaluated individually). Both of them were graded by using a 4-point Likert-like scale (1 = poor, 2 = fair, 3 = good, 4 = excellent) (Supplementary materials, Table 1) [11, 18].

**Fig. 1:**
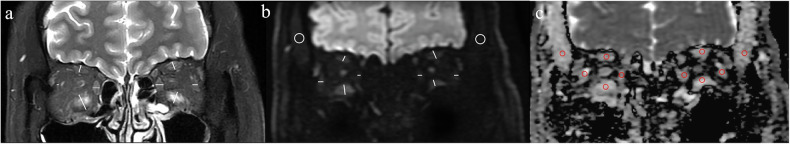
The methods for measurements of GDR, SNR, ADC and nADC values. Coronal T2WI (**a**), coronal FOCUS-MUSE DWI (**b**; *b* = 1000 s/mm^2^) and corresponding ADC image (**c**) in a 32-year-old female with active TAO. For the quantitative measurement of GDR (**a**, **b**), the horizontal diameters of the medial and lateral EOMs and the vertical diameters of the superior and lateral EOMs were measured; For the quantitative measurement of SNR (b), circular ROIs (white, 5 mm^2^) were placed in bilateral temporal muscle, respectively; For the quantitative measurement of ADC and nADC values (c), circular ROIs (red, 2 mm^2^) were placed in EOMs and temporal muscles, respectively. GDR, geometric distortion ratio; SNR, signal-to-noise ratio; ADC, apparent diffusion coefficient; nADC, normalized apparent diffusion coefficient; T2WI, T2-weighted imaging; DWI, diffusion-weighted imaging; TAO, thyroid-associated ophthalmopathy; EOMs, extraocular muscles; ROIs: region of interests

Quantitative measurements of DWI sequences include: (1) geometric distortion ratio (GDR): The horizontal diameters of the medial and lateral EOMs and the vertical diameters of the superior and lateral EOMs were measured and recorded on coronal T2WI and DWI (Fig. 1). The formula is as follows: GDR = (T2WI_diameter of EOM_ – DWI_diameter of EOM_)/T2WI_diameter of EOM_ × 100%; (2) SNR: On the DWI images with uniform SI, a circular region of interest (ROI) (5 mm²) was delineated on the temporalis muscle (Fig. 1). The SI and standard deviation (SD) of the temporalis muscle were recorded, and the SNR was calculated as: SNR = SI/SD. (3) ADC value and normalized ADC (nADC) value: On the same coronal plane of ADC images, circular ROIs (2 mm²) were placed on superior, inferior, medial and lateral EOMs, locating the best visualized section (usually the third slice) behind the eyeball. A circular ROI of the same size was placed at the ipsilateral temporalis muscle (Fig. 1). The ADC values of the four EOMs for both eyes and temporalis muscles were recorded. The nADC value was calculated as follows: nADC = ADC_EOM_/ADC_ipsilateral temporal muscle_. Additionally, the mean ADC values of the four EOMs were calculated, along with the mean nADC values. The unit of ADC values is × 10^-3^mm^2^/s.

Two radiologists (with 6 and 9 years of experience in head and neck radiology) independently evaluated the qualitative parameters and delineated the ROIs. Both of them were blinded to the study design, acquisition parameters, and clinical information. The measurements from the two observers were used to evaluate inter-observer agreement. The measurements of the senior radiologist (with 9 years of experience in head and neck radiology) were used for further analysis.

### Statistical analysis

The Kolmogorov–Smirnov test was used to assess the normality of the distribution. Data with normal distribution were presented as mean ± SD, while non-normally distributed data were expressed as median (25th, 75th percentiles). Differences of qualitative parameters were compared using the Friedman test with the post-hoc Dunn–Bonferroni method among FOCUS-MUSE DWI, MUSE DWI, and FOCUS DWI groups. To compare the quantitative parameters among three groups, the Kruskal-Wallis test with the post-hoc Dunn–Bonferroni method was applied. Independent-samples *t*-test or Mann–Whitney *U-*test were used to evaluate the nADC values of different DWI sequences between active and inactive phases. Receiver operating characteristic (ROC) curves and the DeLong test were used to evaluate the diagnostic efficiency of significant parameters. The corresponding area under the curve (AUC), sensitivity, and specificity for diagnosing active TAOs were also calculated. Inter-observer agreements of qualitative and quantitative parameters were assessed by the kappa coefficient (*κ*) and intraclass correlation coefficient (ICC) analyses, respectively. The κ and ICC values were defined as follows: < 0.40, poor; 0.41–0.60, moderate; 0.61–0.80, good; ≥ 0.81, excellent. All statistical analyses were carried out in SPSS software package (v. 23.0; IBM) and MedCalc (v. 19.3; MedCalc Software bvba). A two-sided *p*-value less than 0.05 was considered statistically significant.

## Results

### Inter-observer assessment of qualitative and quantitative parameters

Inter-observer reproducibility for the qualitative and quantitative parameters of FOCUS-MUSE DWI was good to excellent (*κ*, 0.714–0.873; ICC, 0.615–0.895), while that for FOCUS DWI ranged from moderate to excellent (*κ*, 0.516–0.872; ICC, 0.596–0.869). Meanwhile, moderate to excellent and good to excellent inter-observer reproducibility were obtained when assessing qualitative (*κ*, 0.526–0.872) and quantitative (ICC, 0.627–0.871) parameters for MUSE DWI, respectively. Detailed *κ* and ICC values were shown in Table 2.

**Table 2:** Inter-observer reproducibility for qualitative and quantitative parameters

	FOCUS-MUSE	MUSE	FOCUS
**Qualitative parameters**	***κ***		
Artifacts and geometric distortion	0.783 (0.000–1.000)	0.783 (0.000–1.000)	0.516 (0.071–1.000)
Overall image quality	0.714 (0.000–1.000)	0.783 (0.000–1.000)	0.762 (0.294–1.000)
Sharpness of boundaries			
Right eye			
medial EOM	0.843 (0.000–1.000)	0.634 (0.000–1.000)	0.783 (0.000–1.000)
inferior EOM	0.773 (0.294–1.000)	0.841 (0.000–1.000)	0.841 (0.000–1.000)
lateral EOM	0.870 (0.474–1.000)	0.839 (0.366–1.000)	0.574 (0.000–1.000)
superior EOM	0.873 (0.474–1.000)	0.783 (0.000–1.000)	0.526 (0.000–1.000)
Left eye			
medial EOM	0.873 (0.474–1.000)	0.526 (0.000–1.000)	0.783 (0.000–1.000)
inferior EOM	0.786 (0.000–1.000)	0.872 (0.483–1.000)	0.872 (0.483–1.000)
Lateral EOM	0.870 (0.474–1.000)	0.839 (0.000–1.000)	0.651 (0.000–1.000)
superior EOM	0.858 (0.492–1.000)	0.574(0.000–1.000)	0.651 (0.000–1.000)
**Quantitative parameters**	**ICC**		
GDR			
Right eye			
medial EOM	0.615 (0.191–0.817)	0.627 (0.216–0.822)	0.623 (0.208–0.821)
inferior EOM	0.772 (0.507–0.894)	0.734 (0.416–0.879)	0.596 (0.113–0.816)
lateral EOM	0.662 (0.207–0.820)	0.706 (0.383–0.860)	0.647 (0.259–0.832)
superior EOM	0.673 (0.313–0.844)	0.748 (0.471–0.880)	0.650 (0.265–0.833)
Left eye			
medial EOM	0.640 (0.244–0.829)	0.731 (0.435–0.872)	0.623 (0.207–0.820)
inferior EOM	0.852 (0.681–0.932)	0.734 (0.407–0.881)	0.639 (0.196–0.838)
lateral EOM	0.631 (0.225–-0.825)	0.666 (0.298–0.841)	0.781 (0.541–0.896)
superior EOM	0.700 (0.371–0.857)	0.821 (0.623–0.915)	0.740 (0.453–0.876)
SNR			
right temporal muscle	0.714 (0.400–0.864)	0.767 (0.510–0.889)	0.846 (0.677–0.927)
left temporal muscle	0.704 (0.378–0.859)	0.650 (0.268–0.834)	0.699 (0.369–0.857)
ADC values of EOMs			
Right eye			
medial EOM	0.840 (0.663–0.924)	0.780 (0.537–0.895)	0.854 (0.692–0.930)
inferior EOM	0.643 (0.250–0.830)	0.763 (0.501–0.887)	0.791 (0.560–0.900)
lateral EOM	0.895 (0.779–0.950)	0.834 (0.650–0.921)	0.863 (0.712–0.935)
superior EOM	0.711 (0.393–0.863)	0.788 (0.554–0.899)	0.793 (0.566–0.902)
Left eye			
medial EOM	0.770 (0.517–0.891)	0.871 (0.729–0.939)	0.869 (0.724–0.938)
inferior EOM	0.777 (0.532–0.894)	0.770 (0.517–0.891)	0.861 (0.708–0.934)
lateral EOM	0.767 (0.511–0.889)	0.840 (0.663–0.924)	0.826 (0.635–0.917)
superior EOM	0.721 (0.413–0.867)	0.844 (0.672–0.926)	0.753 (0.480–0.882)
ADC value of right temporal muscle	0.882 (0.753–0.944)	0.712 (0.394–0.863)	0.643 (0.250–0.830)
ADC value of left temporal muscle	0.811 (0.603–0.910)	0.808 (0.597–0.909)	0.830 (0.642–0.919)

*EOM* extraocular muscle, *GDR* geometric distortion ratio, *SNR* signal-to-noise ratio, *ADC* apparent diffusion coefficient, *κ* kappa coefficient, *ICC* intraclass correlation coefficient

### Qualitative parameters among the three DWI groups

**Fig. 2:**
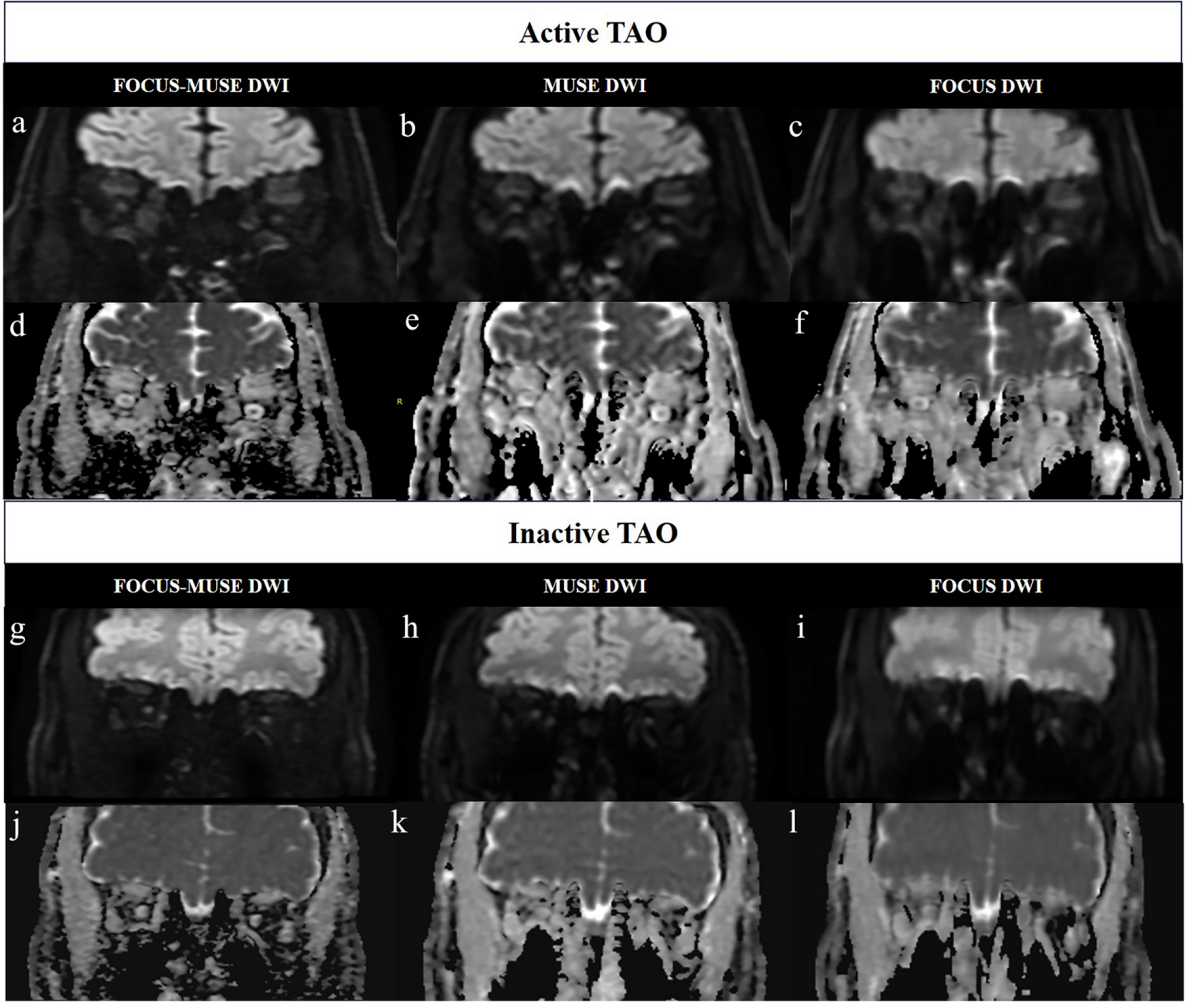
Representative images with FOCUS-MUSE DWI (**a**, **g**; *b* = 1000 s/mm^2^), MUSE DWI (**b**, **h**; *b* = 1000 s/mm^2^), FOCUS DWI (**c**, **i**; *b* = 1000 s/mm^2^) and corresponding ADC images (**d**, **j**; **e**, **k**; **f**, **l**) of an active (male, 71-year-old, CAS = 5) and an inactive (male, 36-year-old, CAS = 2) patients with TAO, respectively. FOCUS-MUSE DWI showed fewer artifacts and geometric distortion, superior image quality, and enhanced sharpness of boundaries compared to MUSE DWI and FOCUS DWI. For active TAO patients, the mean nADC values of left and right eyes were 1.26 and 1.14, respectively. For an inactive TAO patient, the mean nADC values of the left and right eyes were 0.90 and 0.97, respectively. DWI, diffusion-weighted imaging; ADC, apparent diffusion coefficient; TAO, thyroid-associated ophthalmopathy. nADC, normalized apparent diffusion coefficient.

There were significant differences in artifacts and geometric distortion, overall image quality and sharpness of boundaries among the three DWI groups (all *p* < 0.001) (Table 3 and Fig. 2). FOCUS-MUSE DWI exhibited significantly higher scores for artifacts and geometric distortion, overall image quality, and boundary sharpness compared to both MUSE DWI and FOCUS DWI (all *p* < 0.001). MUSE DWI showed significantly higher scores of artifacts and geometric distortion, overall image quality, as well as boundary sharpness in the lateral and superior EOMs of both eyes compared to FOCUS DWI (all *p* < 0.001), while no significant differences were observed for boundary sharpness in other EOMs (all *p* > 0.05) (Table 3).

**Table 3:** Comparisons of qualitative and quantitative parameters among FOCUS-MUSE DWI, MUSE DWI and FOCUS DWI

	FOCUS-MUSE	MUSE	FOCUS	*p*			
				FOCUS-MUSE vs. FOCUS vs. MUSE	FOCUS-MUSE vs. MUSE	FOCUS-MUSE vs. FOCUS	FOCUS vs. MUSE
**Qualitative parameters**							
Artifacts and geometric distortion	3.94 ± 0.24	2.94 ± 0.24	2.13 ± 0.34	< 0.001	< 0.001	< 0.001	< 0.001
Overall image quality	3.93 ± 0.27	2.94 ± 0.24	2.13 ± 0.34	< 0.001	< 0.001	< 0.001	< 0.001
Sharpness of boundaries							
Right eye							
medial EOM	3.03 ± 0.35	2.13 ± 0.34	2.07 ± 0.27	< 0.001	< 0.001	< 0.001	1.000
inferior EOM	2.82 ± 0.49	1.94 ± 0.34	1.91 ± 0.29	< 0.001	< 0.001	< 0.001	1.000
lateral EOM	3.91 ± 0.29	2.90 ± 0.31	2.09 ± 0.34	< 0.001	< 0.001	< 0.001	< 0.001
superior EOM	3.90 ± 0.35	2.93 ± 0.27	2.09 ± 0.29	< 0.001	< 0.001	< 0.001	< 0.001
Left eye							
medial EOM	3.01 ± 0.37	2.13 ± 0.34	2.06 ± 0.30	< 0.001	< 0.001	< 0.001	1.000
inferior EOM	2.84 ± 0.48	1.91 ± 0.34	1.93 ± 0.32	< 0.001	< 0.001	< 0.001	1.000
lateral EOM	3.90 ± 0.31	2.91 ± 0.29	2.06 ± 0.24	< 0.001	< 0.001	< 0.001	< 0.001
superior EOM	3.87 ± 0.39	2.93 ± 0.27	2.06 ± 0.24	< 0.001	< 0.001	< 0.001	< 0.001
**Quantitative parameters**							
GDR							
Right eye							
medial EOM	0.22 ± 0.10	0.37 ± 0.14	0.34 ± 0.15	< 0.001	< 0.001	< 0.001	0.092
inferior EOM	0.30 ± 0.13	0.48 ± 0.13	0.43 ± 0.13	< 0.001	< 0.001	< 0.001	0.003
lateral EOM	0.19 ± 0.10	0.30 ± 0.13	0.25 (0.19, 0.36)	< 0.001	< 0.001	< 0.001	0.230
superior EOM	0.20 ± 0.13	0.33 ± 0.13	0.28 ± 0.13	< 0.001	< 0.001	<0.001	0.015
Left eye							
medial EOM	0.22 ± 0.13	0.37 ± 0.15	0.33 ± 0.14	< 0.001	< 0.001	< 0.001	0.392
inferior EOM	0.35 ± 0.11	0.53 ± 0.10	0.48 ± 0.11	< 0.001	< 0.001	< 0.001	0.024
lateral EOM	0.19 ± 0.12	0.31 ± 0.13	0.26 ± 0.12	< 0.001	< 0.001	0.003	0.001
superior EOM	0.22 ± 0.12	0.34 ± 0.14	0.30 ± 0.15	< 0.001	< 0.001	< 0.001	0.092
SNR							
right temporal muscle	12.60 (11.58, 13.90)	9.01 ± 1.16	9.71 (8.84, 10.48)	< 0.001	< 0.001	< 0.001	0.017
left temporal muscle	12.67 ± 1.66	8.92 ± 1.04	9.69 ± 1.26	< 0.001	< 0.001	< 0.001	0.017
ADC values of EOMs							
medial EOM	1.35 ± 0.19	1.49 ± 0.19	1.43 ± 0.17	< 0.001	< 0.001	< 0.001	0.003
inferior EOM	1.40 (1.29, 1.52)	1.52 (1.42, 1.64)	1.49 ± 0.20	< 0.001	< 0.001	< 0.001	0.002
lateral EOM	1.48 ± 0.17	1.60 ± 0.20	1.55 ± 0.21	< 0.001	< 0.001	0.002	< 0.001
superior EOM	1.43 ± 0.11	1.49 ± 0.15	1.45 (1.33, 1.55)	0.027	0.021	0.648	0.439
mean ADC	1.40 (1.34, 1.48)	1.53 ± 0.15	1.46 (1.39, 1.57)	< 0.001	< 0.001	< 0.001	0.028
nADC values of EOMs							
medial EOM	1.02 (0.94, 1.11)	1.06 ± 0.11	1.05 ± 0.14	0.223	-	-	-
inferior EOM	1.05 (0.99, 1.15)	1.10 ± .012	1.09 ± 0.17	0.097	-	-	-
lateral EOM	1.12 (1.03, 1.19)	1.14 ± 0.13	1.13 ± 0.17	0.223	-	-	-
superior EOM	1.10 ± 0.12	1.04 (0.98, 1.14)	1.07 (0.95, 1.14)	0.028	0.060	0.064	1.000
mean nADC	1.07 (1.01, 1.12)	1.09 ± 0.09	1.08 ± 0.13	0.490	-	-	-

The unit of ADC values: × 10^−3^mm^2^/s

*DWI* diffusion-weighted imaging, *EOM* extraocular muscle, *GDR* geometric distortion ratio, *SNR* signal-to-noise ratio, *ADC* apparent diffusion coefficient, *nADC* normalized apparent diffusion coefficient

### Quantitative parameters between the three groups

There were significant differences in GDR and SNR among the three DWI groups (all *p* < 0.001) (Table 3 and Fig. 2). FOCUS-MUSE DWI showed significantly lower GDR and higher SNR compared to both MUSE DWI and FOCUS DWI (all *p* < 0.05). Muse DWI showed significantly higher GDR in the inferior and superior EOMs of the right eyes, and in the inferior and lateral EOMs of the left eyes, than FOCUS DWI (all *p* < 0.05). The SNR of MUSE DWI were significantly lower than FOCUS DWI (all *p* < 0.05).

No significant differences were observed in the ADC or nADC values of the FOCUS-MUSE DWI between the left and right eyes (all *p* > 0.05). In contrast, the ADC values of the lateral EOMs in both MUSE and FOCUS DWIs, as well as the nADC values of the lateral EOMs in MUSE DWI, showed significant inter-eye differences (all *p* < 0.05) (Supplementary materials, Table 2).

Each eye of every patient was treated as an independent data point. There were also significant differences in ADC values of EOMs and their mean value among the three DWI groups (all *p* < 0.05) (Table 3 and Fig. 2). ADC values of EOMs and their mean value were generally lower in FOCUS-MUSE DWI than those in MUSE DWI (all *p* < 0.05) and those in FOCUS DWI (all *p* < 0.05, except for that of superior EOM). MUSE DWI showed significantly higher ADC values of EOMs and their mean value than FOCUS DWI (all *p * < 0.05, except for that of the superior EOM). As for the nADC values of EOMs and their mean value, no significance was observed among the three DWI groups (all *p* > 0.05), except for that of the superior EOM (*p* = 0.028) (Table 3). However, the adjusted P values of superior EOM were not statistically different between any two groups (all *p* > 0.05).

### Comparisons of nADC values between active and inactive groups

**Fig. 3:**
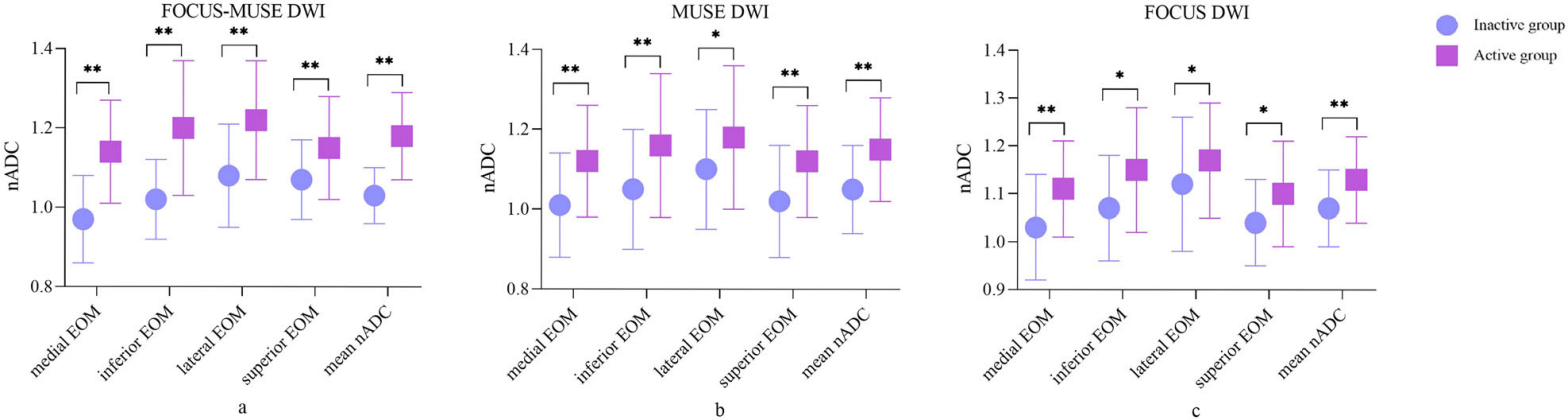
Comparisons of nADC values between active and inactive TAOs (** *p* < 0.001, * *p* < 0.05). nADC, normalized apparent diffusion coefficient; TAO, thyroid-associated ophthalmopathy; EOM, extraocular muscle; DWI, diffusion-weighted imaging

Active TAOs showed significantly higher nADC values across all EOMs and higher mean nADC values compared to inactive TAOs in FOCUS-MUSE DWI, MUSE DWI, and FOCUS DWI (all *p* < 0.05). Detailed results regarding the comparisons of nADC values between active and inactive TAOs were shown in Table 4 and Fig. 3.

**Table 4:** Comparisons of nADC values of EOMs in FOCUS-MUSE DWI, MUSE DWI, and FOCUS DWI between the active and inactive TAO groups

	Inactive group	Active group	*p*
FOCUS-MUSE			
medial EOM	0.97 ± 0.11	1.14 ± 0.13	< 0.001
inferior EOM	1.02 ± 0.10	1.20 ± 0.17	< 0.001
lateral EOM	1.08 ± 0.13	1.22 ± 0.15	< 0.001
superior EOM	1.07 ± 010	1.15 ± 0.13	< 0.001
mean nADC	1.03 ± 0.07	1.18 ± 0.11	< 0.001
MUSE			
medial EOM	1.01 ± 0.13	1.12 ± 0.14	< 0.001
inferior EOM	1.05 ± 0.15	1.16 ± 0.18	< 0.001
lateral EOM	1.10 ± 0.15	1.18 ± 0.18	0.015
superior EOM	1.02 ± 0.14	1.12 ± 0.14	< 0.001
mean nADC	1.05 ± 0.11	1.15 ± 0.13	< 0.001
FOCUS			
medial EOM	1.03 ± 0.11	1.11 ± 0.10	< 0.001
inferior EOM	1.07 ± 0.11	1.15 ± 0.13	0.002
lateral EOM	1.12 ± 0.14	1.17 ± 0.12	0.031
superior EOM	1.04 ± 0.09	1.10 ± 0.11	0.001
mean nADC	1.07 ± 0.08	1.13 ± 0.09	< 0.001

*nADC* normalized apparent diffusion coefficient, *EOM* extraocular muscle, *DWI* diffusion-weighted imaging, *TAO* thyroid-associated ophthalmopathy

### Diagnostic performance of nADC values for active TAO in different DWI sequences

**Fig. 4:**
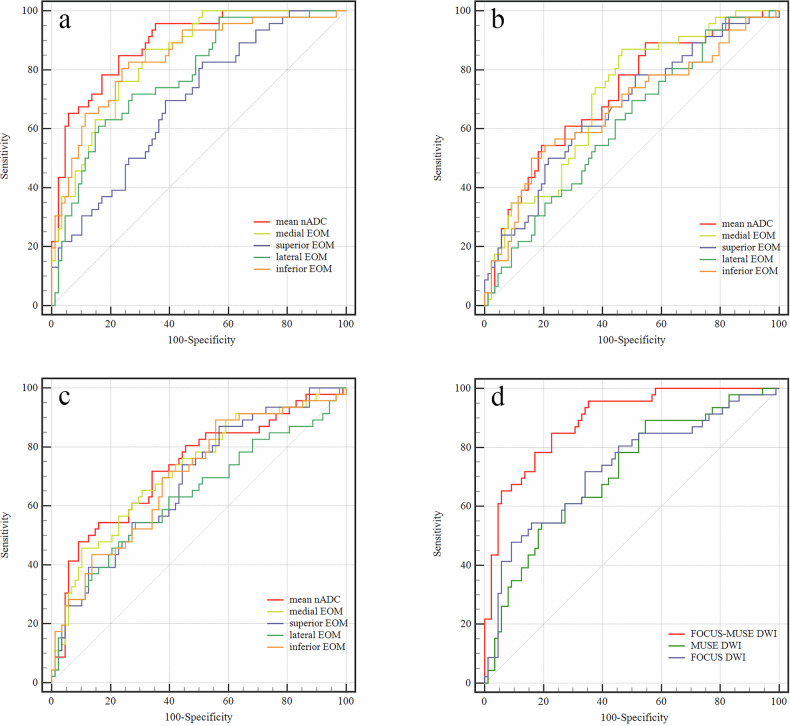
Receiver operating characteristic curves of nADC values of EOMs and mean nADC values for discriminating active TAO based on FOCUS-MUSE DWI (**a**), MUSE DWI (**b**), and FOCUS DWI (**c**), respectively. Receiver operating characteristic curves of mean nADC values among three sequences for discriminating active TAO (**d**). nADC, normalized apparent diffusion coefficient; EOM, extraocular muscle; TAO, thyroid-associated ophthalmopathy; DWI, diffusion-weighted imaging

Within a single DWI sequence, comparisons consistently demonstrated that the AUC of the mean nADC value of EOMs exceeded that of the single EOM. Comparisons of diagnostic performance across the three DWI groups indicated that, mean nADC value of EOMs in FOCUS-MUSE DWI exhibited the highest AUC for diagnosing active TAOs (AUC, 0.890; cut-off value, 1.08; sensitivity, 84.8%; specificity, 77.3%), followed by that in FOCUS DWI (AUC, 0.730; cut-off value, 1.17; sensitivity, 47.8%; specificity, 90.9%; *p* < 0.001), and that in MUSE DWI (AUC, 0.713; cut-off value, 1.13; sensitivity, 54.3%; specificity, 80.7%; *p* < 0.001) (Table 5 and Fig. 4).

**Table 5:** Diagnostic performance of nADC values of EOMs for staging TAO

	AUC	Cut-off	Sensitivity%	Specificity%
FOCUS-MUSE				
medial EOM	0.842	1.01	87.0	68.2
Inferior EOM	0.841	1.09	80.4	76.1
lateral EOM	0.780	1.17	60.9	84.1
superior EOM	0.692	1.05	82.6	48.9
mean nADC	0.890	1.08	84.8	77.3
MUSE				
medial EOM	0.708	1.01	87.0	53.4
inferior EOM	0.668	1.18	50.0	84.1
lateral EOM	0.614	1.12	69.6	50.0
superior EOM	0.669	1.11	50.0	78.4
mean nADC	0.713	1.13	54.3	80.7
FOCUS				
medial EOM	0.723	1.16	45.7	89.8
inferior EOM	0.698	1.00	89.1	44.3
lateral EOM	0.636	1.19	54.3	71.6
superior EOM	0.684	1.00	87.0	43.2
mean nADC	0.730	1.17	47.8	90.9

*nADC* normalized apparent diffusion coefficient, *EOM* extraocular muscle, *TAO* thyroid-associated ophthalmopathy

## Discussion

Our study illuminated two main findings. First, among the three EPI-based modified DWI sequences, FOCUS-MUSE DWI demonstrated superior image quality than MUSE DWI and FOCUS DWI, as assessed through comparisons of artifacts and geometric distortion, overall image quality, sharpness of boundaries, SNR and GDR. Second, the mean nADC value of EOMs obtained from FOCUS-MUSE DWI demonstrated the highest diagnostic performance for discriminating the activity of TAO. Our findings may offer valuable insights into the benefits of FOCUS-MUSE DWI relative to MUSE DWI and FOCUS DWI in the context of TAO and other orbital diseases.

Our study found that FOCUS-MUSE DWI outperformed both MUSE DWI and FOCUS DWI in terms of image quality, offering fewer artifacts and geometric distortion, superior image quality, enhanced sharpness of boundaries, higher SNR and lower GDR, consistent with the study of Bai et al, who evaluated the reliability of FOCUS-MUSE DWI compared to FOCUS and MUSE DWIs in the pancreas [11]. FOCUS DWI enhances spatial resolution and SNR by reducing the FOV in the phase-encoding direction, thereby mitigating geometric distortions and artifacts [12, 19]. MUSE DWI, through multishot excitation and segmented k-space acquisition along the phase-encoding axis, improves image detail retention and reduces motion artifact susceptibility [13, 15]. Thus, the integration of both techniques in FOCUS-MUSE DWI logically results in enhanced image quality [11].

In our study, the ADC values derived from FOCUS-MUSE DWI were significantly lower than those obtained from MUSE DWI. Furthermore, the ADC values of EOMs (except for the superior EOM) and the mean ADC value in FOCUS-MUSE DWI were lower than those in FOCUS DWI. Ilma et al demonstrated that ADC values are influenced by multiple factors, including noise floors, the amount of T1 and mainly T2 weighting in the tissue [20]. Besson et al demonstrated that correcting susceptibility distortions enhances the accuracy of ADC quantification [21]. Therefore, it is reasonable to infer that the superior image quality of FOCUS-MUSE DWI in this study may enhance the accuracy of ADC value measurements. Moreover, good to excellent inter-observer reproducibility of FOCUS-MUSE DWI was obtained in our study. Thus, the ADC values obtained from FOCUS-MUSE DWI demonstrate sufficient reliability and repeatability for the diagnosis of orbital diseases.

In recent years, some studies have demonstrated that ADC values could be used to evaluate the disease activity and the treatment response for TAO [22–25]. To be more objective, we used nADC values, which narrowed the differences among the three groups, to evaluate TAO activity rather than absolute ADC values. In accordance with previous studies, we found the nADC values of active TAO patients were significantly higher than those in the inactive phase [22, 24]. This may be propelled by distinct pathological changes such as edema of EOMs in the active phase and fibrosis or fatty infiltration of EOMs in the inactive phase [25]. Further ROC results indicated that the mean nADC values of FOCUS-MUSE DWI showed optimal diagnostic performance with the highest AUC compared to MUSE DWI and FOCUS DWI (0.890 vs 0.713, 0.730). Based on the findings of the current study, FOCUS-MUSE DWI outperformed the other two EPI-based modified DWIs in orbital applications, exhibiting superior image quality and diagnostic accuracy in assessing TAO activity. We recommend FOCUS-MUSE DWI for clinical use, as it offers enhanced capabilities for disease activity detection and staging.

This study has several limitations. First, the study cohort is relatively small. Larger-scale studies are required to validate our findings. Second, only EPI-based modified DWI sequences were used. Future research incorporating additional imaging modalities may enhance the diagnostic efficiency for active TAO.

In conclusion, FOCUS-MUSE DWI offers optimal orbital image quality and staging performance for TAO compared to MUSE DWI and FOCUS DWI. We suggest using FOCUS-MUSE DWI to evaluate patients with TAO in daily practice.

## Supplementary information

ELECTRONIC SUPPLEMENTARY MATERIAL

## Data Availability

The data that support the findings of this study are not openly available due to reasons of sensitivity and are available from the corresponding author upon reasonable request.

## Abbreviations

ADC: Apparent diffusion coefficient
AUC: Area under the curve
CAS: Clinical activity score
DWI: Diffusion-weighted imaging
EOMs: Extraocular muscles
FOCUE-MUSE: Field-of-view optimized and constrained undistorted single-shot multiplexed sensitivity-encoding
FOCUS: Field-of-view optimized and constrained undistorted single-shot
FOV: Field-of-view
GDR: Geometric distortion ratio
ICC: Intraclass correlation coefficient
*κ*: Kappa coefficient
MUSE: Multiplexed sensitivity-encoding
nADC: Normalized ADC
ROC curve: Receiver operating characteristic curve
ROI: Region of interest
SI: Signal intensity
SNR: Signal-to-noise ratio
SS-EPI: Single-shot echo-planar imaging
SD: Standard deviation
TAO: Thyroid-associated ophthalmopathy
TE: Echo time
TR: Repetition time
