# Supplementary material for: FOCUS-MUSE DWI outperforms MUSE and FOCUS DWIs in orbital imaging quality and staging thyroid-associated ophthalmopathy
Source: Insights Imaging. 2025 Nov 6;16:246. doi: 10.1186/s13244-025-02129-9 (PMC12592608; doi:10.1186/s13244-025-02129-9)
Supplement: Supplementary file 1 — ELECTRONIC SUPPLEMENTARY MATERIAL [file 13244_2025_2129_MOESM1_ESM.pdf]

**FOCUS-MUSE DWI outperforms MUSE and FOCUS DWIs in orbital imaging quality and staging thyroid-associated ophthalmopathy**

**ELECTRONIC SUPPLEMENTARY MATERIAL**

Table 1. Image quality scoring criteria of qualitative parameters

| Qualitative parameters | Score |
|---|---|
| Artifacts and geometric distortion | |
| Severe artifacts or geometric distortions that significantly impair structural representation | 1 |
| Moderate artifacts geometric distortion that partially affect structural representation | 2 |
| Mild artifacts or geometric distortion that have no impact on structural representation | 3 |
| No artifacts or geometric distortion | 4 |
| Overall image quality | |
| Poor image quality | 1 |
| Fair image quality | 2 |
| Good image quality | 3 |
| Excellent image quality | 4 |
| Sharpness of boundaries | |
| Anatomical structures are poorly delineated | 1 |
| The anatomical structure is well-defined, though with blurred edges | 2 |
| The anatomical structures are well visualized, with slightly blurred edges | 3 |
| The anatomy is distinctly clear, with sharp delineation of edges | 4 |

Table 2. Comparisons of ADC values and nADC values between left and right eyes of TAO patients.

| | Right eyes | Left eyes | P |
|---|---|---|---|
| | ***ADC values*** | | |
| FOCUS-MUSE | | | |
| medial EOM | 1.37±0.18 | 1.34±0.19 | 0.344 |
| inferior EOM | 1.43±0.18 | 1.40±0.19 | 0.352 |
| lateral EOM | 1.49±0.17 | 1.46±0.18 | 0.300 |
| superior EOM | 1.42 (1.38, 1.47) | 1.43±0.11 | 0.855 |
| MUSE | | | |
| medial EOM | 1.49±0.19 | 1.47±0.19 | 0.483 |
| inferior EOM | 1.57±0.19 | 1.52±0.18 | 0.149 |
| lateral EOM | 1.65±0.18 | 1.55±0.20 | **0.003** |
| superior EOM | 1.51±0.16 | 1.42 (1.36, 1.54) | 0.066 |
| FOCUS | | | |
| medial EOM | 1.44±0.18 | 1.43±0.16 | 0.698 |
| inferior EOM | 1.52±0.21 | 1.46±0.20 | 0.148 |
| lateral EOM | 1.59±0.18 | 1.51±0.23 | **0.041** |
| superior EOM | 1.47±0.18 | 1.42±0.17 | 0.115 |
| | ***nADC values*** | | |
| FOCUS-MUSE | | | |
| medial EOM | 1.03±0.15 | 1.03±0.15 | 0.852 |
| inferior EOM | 1.05 (0.99, 1.15) | 1.08 (0.99, 1.16) | 0.970 |
| lateral EOM | 1.12 (1.03, 1.18) | 1.13±0.15 | 0.920 |
| superior EOM | 1.07 (1.00, 1.14) | 1.11±0.11 | 0.124 |
| mean nADC | 1.08 (1.01, 1.13) | 1.07 (1.01, 1.13) | 0.959 |
| MUSE | | | |
| medial EOM | 1.06±0.11 | 1.05±0.12 | 0.704 |
| inferior EOM | 1.11±0.12 | 1.09±0.12 | 0.264 |
| lateral EOM | 1.17±0.12 | 1.11±0.14 | **0.006** |
| superior EOM | 1.07±0.10 | 1.05±0.09 | 0.213 |
| mean nADC | 1.10±0.09 | 1.08±0.09 | 0.062 |
| FOCUS | | | |
| medial EOM | 1.04±0.14 | 1.06±0.14 | 0.513 |
| inferior EOM | 1.09±0.16 | 1.08±0.17 | 0.690 |
| lateral EOM | 1.15±0.16 | 1.11±0.17 | 0.279 |
| superior EOM | 1.08 (0.97, 1.13) | 1.05±0.15 | 0.931 |
| mean nADC | 1.08±0.13 | 1.07±0.13 | 0.671 |

The unit of ADC values: × $10^{-3}mm^2/sec$.

ADC, apparent diffusion coefficient; nADC, normalized apparent diffusion coefficient; TAO, thyroid-associated ophthalmopathy; EOM, extraocular muscle.
